# Effects of *Momordica charantia *L. on the Blood Rheological Properties in Diabetic Patients

**DOI:** 10.1155/2014/840379

**Published:** 2014-02-03

**Authors:** Eduardo Luzía França, Elton Brito Ribeiro, Edson Fredulin Scherer, Déborah Giovanna Cantarini, Rafael Souza Pessôa, Fernando Luzía França, Adenilda Cristina Honorio-França

**Affiliations:** ^1^Institute of Biological and Health Science, Federal University of Mato Grosso (UFMT), Rodovia BR070, Km 5 s/n**º**, 78600-000 Barra do Garças-MT, Brazil; ^2^Federal University of Ouro Preto, Ouro Preto, MG, Brazil

## Abstract

An evaluation of the rheological properties and the effects of *Momordica. charantia *L. (*M. charantia*) nanoparticles and polyethylene glycol (PEG) microspheres adsorbed with *M. charantia* nanoparticles on the blood of hyperglycemic patients is presented. Blood samples were collected according to glycemic status: normoglycemic (*N* = 56) and hyperglycemic (*N* = 26). General and hematological characteristics were determined. Blood rheological parameters were determined at room temperature and under a temperature scan. We determined the effects on whole blood viscosity of treatment with an extract of *M. charantia*, PEG, or PEG microspheres adsorbed with plant extract. The viscosity of the blood of hyperglycemic patients is greater than that of normoglycemic patients. Nanoparticles of *M. charantia* extracts lowered blood viscosity at equivalent rates in normo- and hyperglycemic individuals. PEG microspheres did not reduce blood viscosity in hyperglycemic individuals. However, PEG microspheres adsorbed with nanofraction extracts of *M. charantia* reduced blood viscosity. These data suggest that the effects of diabetes on the viscosity of the blood should be considered. The use of a nanoparticles extract of *M. charantia* and its adsorption on PEG microspheres may represent an alternative for the control and treatment of blood disorders in diabetic patients.

## 1. Introduction

Diabetes mellitus is a metabolic disease characterized by elevated blood glucose levels. It results from the absence of, or inadequate levels of, pancreatic insulin secretion, with or without concurrent impairment of insulin action [1].

Diabetic patients have endothelial dysfunction and damage caused to the vessel wall [2]. The hyperglycemia in diabetes is associated with an increased risk for plasma hypercoagulability [3].

The pathophysiology of vascular disease in diabetes involves abnormalities in endothelial, vascular smooth muscle cell and platelet function. The metabolic abnormalities that characterize diabetes, such as hyperglycemia, increased free fatty acids, and insulin resistance, can provoke molecular mechanisms that contribute to vascular dysfunction [4].

Reports in the literature suggest that increased serum osmolarity of the blood occurs in diabetic patients due to high sugar concentrations [5]. In addition, increased capillary permeability and hematocrits imply a possible increase in blood viscosity. These rheological changes may play a role in the pathogenesis of diabetic complications [5, 6].

Red blood cell rheology is altered in different diseases, including diabetes [7]. Rheological studies in diabetes have been designed to determine variables such as blood and plasma viscosity, the aggregation and deformability of erythrocytes, and the deformability of neutrophils [8].

Rheological alterations can be verified by the deformation or aggregation of erythrocytes, blood flow [8–11], and increased spherical shape [7]. Studies have shown that vascular damage is one of the major characteristic of diabetes [12], which is accompanied by rheological abnormalities that can cause hyperviscosity syndrome [13], that is, inadequate metabolic control associated with changing values of blood viscosity [14].

Because of the changes in blood viscosity in the pathogenesis of diabetes, rheology can be an important tool in monitoring patients with diabetes mellitus [6]. Moreover, substances that can reduce blood viscosity in chronic diseases may be a promising way to reduce the metabolic complications of these disorders.

Various experimental studies have shown that plants have the ability to reduce glucose levels [15]. Several medicinal plants display beneficial effects against disease, mainly chronic degenerative diseases such as diabetes [15–17]. Brazilian ethnopharmacological studies of *Momordica charantia* L. (*M. charantia*) showed that it had hypoglycemic properties with a direct insulin mimetic effect [18, 19]. Studies have shown that this plant can repair damage *β*-cells, thereby stimulating insulin levels [19, 20] and improving sensitivity and signaling of insulin [19, 21].

On the other hand, polyethylene glycol (PEG) microspheres are polymeric particles that absorb organic compounds and are used experimentally as a carrier molecule. They can be modified to improve their biological function and are useful for the delivery of a variety of substances or therapeutic plants [22–25]. This system was shown to provide additional protection against infections in hyperglycemic individuals, suggesting that *M. charantia *adsorbed to PEG microspheres could be a tool in the treatment of diabetes [25].

Despite the importance and high incidence of this disorder [26–29], the effects of *M. charantia *nanoparticles and microspheres with adsorbed extracts on rheological changes in hyperglycemic human blood are still not understood. Therefore, the aim of this study was to evaluate the rheological properties and the effects of *M. charantia *nanoparticles and *M. charantia *nanoparticles adsorbed to PEG microspheres in the blood of hyperglycemic patients.

## 2. Materials and Methods

### 2.1. Subjects

Samples of 15 mL of blood were collected from 56 volunteers normoglycemic and 26 volunteers with type II diabetes mellitus, noninsulin dependent without micro- or macroangiopathy. Blood samples were stored in heparinized (25 U mL^−1^) tubes. The volunteers signed an informed consent form that was approved by the Local Ethics Committee before entering the study.

The controlled variables were smoking status (yes/no), arterial hypertension (yes/no), and glycemic index (GI) based on mean plasma glucose level. The GI was classified as normoglycemic (GI < 120 mg dL^−1^) or hyperglycemic (GI ≥ 120 mg dL^−1^).

### 2.2. Glucose Determination

Glucose levels were determined by an enzymatic system. Samples of 10 *μ*L of serum or a standard of 100 mg dL^−1^ (BioTécnica, Varginha, Brazil) were placed in 1.0 mL of phosphate buffer solution (182.42 mmol L^−1^, pH 7.0, with 0.3 mmol L^−1^4-aminoantipyrine, 10 mmol L^−1^ phenol, 15000 U L^−1^ glucose oxidase, and 1200 U L^−1^ peroxidase). The suspensions (samples and standard) were mixed and incubated for 10 min at 37°C. The reactions were read on a spectrophotometer at 505 nm.

### 2.3. Determination of Body Mass Index (BMI), Body Mass, Abdominal Circumference, Age, and Stature

To calculate BMI (mass/stature²) values of body mass (kg) and stature (m) were considered, which were obtained with the assistance of a mechanical balance (capacity to record 120 kg and an accuracy of 0.1 kg) and inelastic tape, respectively. The abdominal circumference was assessed using an inelastic tape.

### 2.4. Preparation of Extract of *M. charantia* L

The plants were collected and deposited in the herbarium at the Institute of Biological and Health Science-Federal University of Mato Grosso, Pontal do Araguaia, MT, Brazil, located at Lat. 15°55′08′′S and Long. 52°16′44′′W at an altitude of 365 m. Preparation of the plants involved a mixing process followed by maceration according to the Brazilian pharmaceutical code [25]. The plants were macerated by placing 200 g of the plant into 1 L of distilled water. After the preparation was filtered, the *M. charantia *L. extract was stored at 4°C.

### 2.5. Poly(ethylene glycol) (PEG) Microspheres Preparation

The microspheres were obtained from Poly(ethylene glycol) (PEG) 6000 (Sigma, ST Louis, USA) using a modification protocol [22, 25]. Briefly, 20 g of PEG 6000 was resuspended in 100 mL of a 2% sodium sulfate solution in phosphate-buffered saline (PBS) and incubated at 37°C for 45 min. After incubation, the PEG microspheres were diluted 3 : 1 in PBS and washed twice in PBS (500 ×g, 5 min). The PEG microspheres were resuspended in PBS. The formation of the microspheres was thermally induced by subjecting the solution to 95°C for 5 min. For adsorption, the suspensions of PEG microspheres in PBS were incubated with *M. charantia* extract (v/v) at 37°C for 30 minutes. The samples were then analyzed by optical microscopy.

### 2.6. Treatment of Blood with *M. charantia*, PEG Microspheres, and PEG Microspheres Adsorbed with *M. charantia*

To assess the effects of *M. charantia* on rheological parameters, 450 *μ*L of human blood was incubated with 150 *μ*L of *M. charantia *(100 ng mL^−1^) for 30 min at 37°C.

To investigate the effects on rheological parameters of PEG microspheres adsorbed with *M. charantia*, blood (450 *μ*L) was incubated with 150 *μ*L of PEG microspheres adsorbed with *M charantia *(v/v) for 30 min at 37°C. For comparison, the effects of PEG microspheres alone on rheological parameters were measured as follows. Samples of blood (450 *μ*L) were incubated with 150 *μ*L of PEG microspheres (v/v) for 30 min at 37°C. The suspensions were then immediately used in the rheological parameter assays.

### 2.7. Blood Rheological Parameters

The rheological parameters were measured using the Modular Compact Rheometer—MCR 102 (Anton Paar GmbH, Ostfildern, Germany). In all experiments, 600 *μ*L of blood (normo- or hyperglycemic) was applied to the surface of the plate reader following the removal of excess sample. The readings were taken with permanent control of gap measurements with TruGap in 0.099 mm increments and the measuring cell Toolmaster CP 50 and precise temperature control using T-Ready and the software Rheoplus V3.61. The graphics were obtained using Rheoplus. For the flow curves and viscosity, established parameters were based on the control of shear stress (*τ*) to 0–5 Pa for upsweep and 5–0 Pa for downward curves. The tests were conducted under isothermal conditions at 37°C, with 75 readings analyzed.

For the viscosity curve under temperature scan, established parameters were based on fixed control shear stress (*τ*) to 1 Pa with a variation of temperature from 25 ± 0.1 to 45 ± 0.1°C and a heating rate of 1°C/min. Parameters were recorded every 0.5 ± 0.1°C, with 41 readings analyzed.

### 2.8. Statistical Analysis

The statistically significant difference was evaluated using the analysis of variance (ANOVA) and the statistical significance was considered for a *P*  value less than 0.05.

## 3. Results

### 3.1. Subject Characteristics

Age, stature, abdominal circumference, body mass, and body mass index were similar between the two groups. The erythrocyte and leukocyte concentrations were similar between normoglycemic and diabetic groups. The glucose concentration was higher in the diabetic group than in the normoglycemic group (Table 1).

### 3.2. Determination of Blood Rheological Parameters

**Figure 1:**
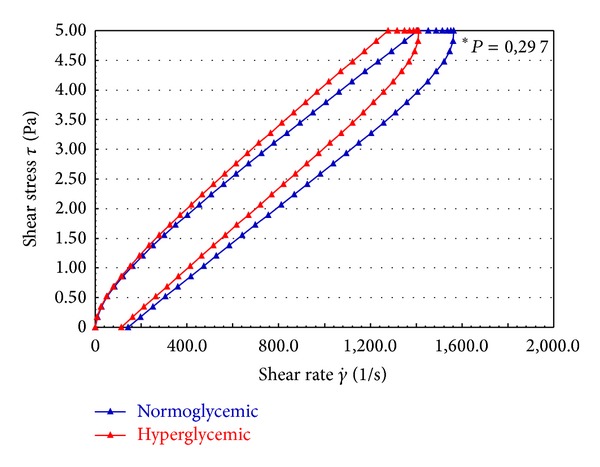
Flow curve of normoglycemic and hyperglycemic whole blood. *No statistical difference (*P* > 0.05).

The rheological profiles of the blood of normo- and hyperglycemic individuals are shown in Figure 1. There were no significant differences in blood flow between the groups. The curve of blood flow in both groups began at the origin, ascended, and was nonlinear (Figure 1).

**Figure 2:**
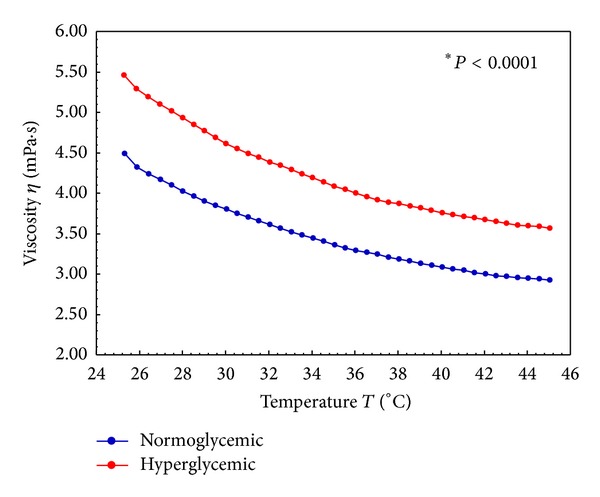
Viscosity curve of normoglycemic and hyperglycemic whole blood under a temperature scan (25 at 45°C). *Statistical difference (*P* < 0.05).

**Figure 3:**
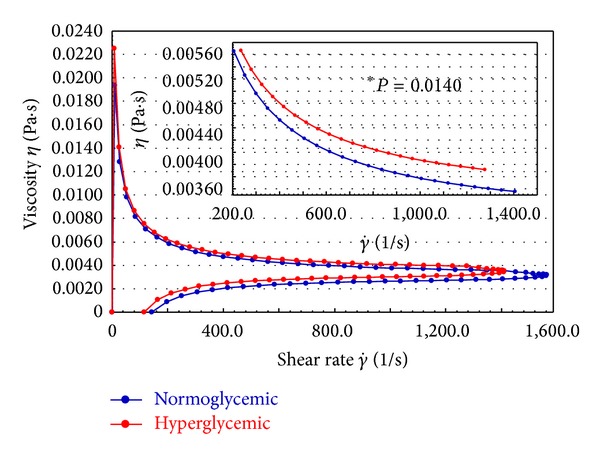
Viscosity curve of normoglycemic and hyperglycemic whole blood. *Amplification of the region with a statistical difference (*P* < 0.05).

In Figures 2 and 3, the blood viscosity data for the normo- and hyperglycemic individuals are shown. The viscosity of the blood was greater in hyperglycemic individuals compared to normal subjects and at different temperatures. There was a gradual and homogeneous reduction, independent of temperature, in the blood viscosity profile in both groups (Figure 2). The analysis of blood viscosity at 37°C revealed that the viscosity of the blood of hyperglycemic patients is significantly greater than that of normoglycemic individuals (Figure 3).

**Figure 4:**
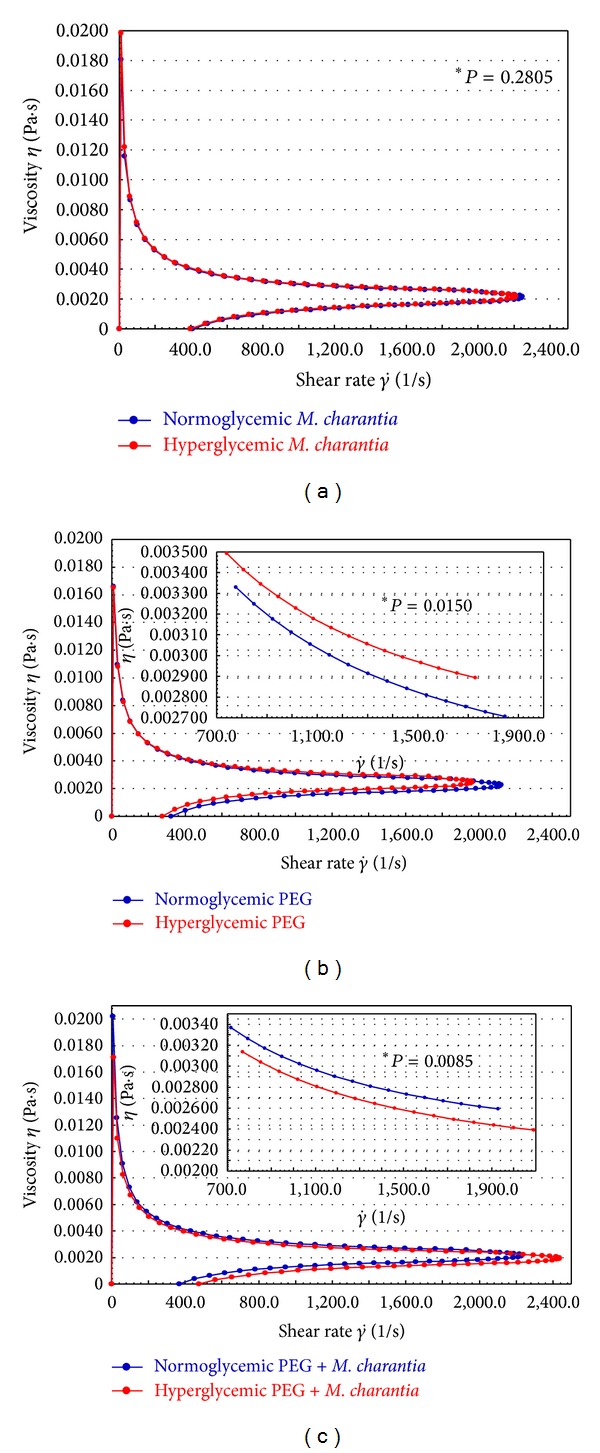
Viscosity curves of normoglycemic and hyperglycemic whole blood after treatment. (a) Treatment with *M. charantia *L. (b) Treatment with PEG microspheres. (c) Treatment with PEG microspheres adsorbed with *M. charantia *L. *Amplification of the region with a statistical difference (*P* < 0.05).

When blood from both groups was treated with *M. charantia, *the blood viscosity was similar between groups. Incubation of hyperglycemic blood with nanoparticles of *M. charantia *extract reduced blood viscosity at rates equivalent to normoglycemic blood (Figure 4(a)).

The effects of PEG microspheres on blood viscosity in the experimental groups are described in Figure 4(b). The viscosity of blood in hyperglycemic individuals incubated with PEG microspheres is significantly greater than in normoglycemic individuals.

The viscosity profile of whole blood from hyperglycemic and normoglycemic patients treated with nanoparticles of *M. charantia* extract adsorbed to PEG microspheres is shown in Figure 4(c). The blood viscosity in hyperglycemic individuals is also reduced in the presence of the PEG microspheres adsorbed with nanoparticles of an extract of *M. charantia,* with viscosity values below the values observed for the normoglycemic group (Figure 4(c)).

The mean viscosities of whole blood and the respective treatment groups are summarized in Table 2. For the conditions of shear stress and rate, the results are similar to those of the viscosity profiles (Figures 3 and 4). It can be seen that the viscosity of the blood of hyperglycemic patients is greater than that of normoglycemic patients, that nanoparticles of the extract of *M. charantia *lower blood viscosity at equivalent rates in normo- and hyperglycemic individuals, and that the PEG microspheres did not reduce the viscosity of the blood of hyperglycemic individuals. However, when adsorbed with nanoparticles of an extract of *M. charantia, *the microspheres reduced viscosity.

## 4. Discussion

This study showed that the blood from hyperglycemic patients exhibits changes in the viscosity and that nanoparticles of an extract of *M. charantia *adsorbed or not to PEG microspheres were able to reduce this viscosity to levels similar to normoglycemic.

The evidence underestimates the overall difference in rheological properties between diabetic and control lymphocytes [30]; therefore, the rheological blood tissue may be explained by the presence of erythrocytes. These corpuscular cells suspended in fluid form are different in the macro- and microcirculatory system, because there are variations in shear in different regions of the circulatory system [10].

In this study, the rheological profile of the blood flow, independent of glycemic levels, does not show the characteristic behavior of ideal liquids. Instead of a straight line, there is a flow curve starting from the origin of the flow. The ascendant and nonlinear behavior starting at the origin characterizes the fluids, nonNewtonian and pseudoplastic, for these shear conditions [31–33]. In the ascending and descending flow, the top curve is not superimposed. There is an area of hysteresis, which defines the magnitude of the property of thixotropy [34].

The lack of superimposition of the ascending and descending curves with an area of hysteresis was observed in this study, demonstrating that thixotropic properties are present in the blood flow. In the literature, study demonstrates that thixotropic behavior in diabetes blood is substantially increased. The diabetic pattern appears produced by a combination of reduced erythrocyte deformability and increased erythrocyte aggregation due to plasma protein changes [35]. There are lines of evidence that the red blood cells in other diseases too increase thixotropic properties of blood [36].

The integrity of this property is important, because it shows that the blood has the potential for reversible deformation in the various conditions of shear that blood flow undergoes [32]. The recovery of blood flow in the tissue depends on structural and functional integrity and body temperature [37].

This work evaluates the influence of temperature on the dynamic variation of the viscosity of blood tissue; it was observed that the blood of hyperglycemic patients has a greater viscosity over a wide temperature range and at physiological temperatures, which can trigger adaptation and deformation difficulties.

Previous studies concluded that untreated children with diabetes show more pronounced alterations of erythrocyte and PMN deformability than insulin-treated diabetic children. High counts of rigid active PMN, impaired deformability of resting PMN, and decreased flexibility of erythrocytes at low flow may all contribute to the high risk that children with diabetes have for acute vascular complications [8].

In the literature, studies of healthy subjects revealed that the rate of elongation (representing the deformability) decreased significantly with temperature reductions of 37°C to 5°C [38]. In this study, increased blood viscosity with decreasing temperature may be one explanation for the decreased deformability. Changes in the mechanical properties of cell membranes are sensitive to temperature. The body temperature can have significant effects on changes in the deformability of red blood cells and, associated with other factors, can cause changes in viscosity [10].

According to the results of recent clinical trials, therapeutic aphaeresis reducing the concentration of *α*_2_-macroglobulin might provide efficient improvement of overall blood fluidity in the microcirculation [39]. There is a reduction of both plasma and whole blood viscosity, which, with a series of treatments, can lead to sustained microcirculatory recovery and significantly change the natural course of a chronic disease [40, 41].

On the other hand, several studies have shown that ethnopharmacological medicinal plants exhibit beneficial effects on various diseases and could be alternative treatments principally in chronic degenerative diseases, such as diabetes [15–17]. The utilization of nanoparticles of *M. charantia* can be useful in clinical applications in diabetics [27]. In this study, the effects of the extract of *M. charantia* on the viscosity of the hyperglycemic blood patients was observed. The treatment with the nanoparticles extract of *M. charantia *determined profiles and mean viscosity of whole blood from hyperglycemic patients similar to the normoglycemic individuals, which indicates that the extract was effective in reducing the viscosity of hyperglycemic blood.

The reduction in the viscosity of hyperglycemic blood by *M. charantia* can be associated with its hypoglycemic properties [15] and its action as an insulin mimetic [18], as well as its ability to affect the immune system [42, 43]. In addition, the erythrocytes of patients with uncontrolled diabetes are more sensitive to osmotic shock and are more exposed to free radicals [6]; studies suggest that the reduction of viscosity by *M. charantia *can also be related to a possible antioxidant activity [18, 44].

The therapeutic properties of compounds isolated from plants and their incorporation into controlled release systems offer an important strategy for developing drugs with intelligent properties. In the literature, the immunomodulatory effects of PEG microspheres adsorbed with *M. charantia* on blood phagocytes suggest that this system may be useful for the delivery of a variety of therapeutic plants. The system was also shown to provide additional protection against infections in hyperglycemic individuals [19]. In this study, we evaluated the effects on viscosity of *M. charantia* associated with PEG microspheres. The PEG microspheres did not change the profile or average viscosity of hyperglycemic whole blood, suggesting that these microspheres do not act directly on the blood in these individuals. However, treatment with a nanofraction extract of *M. charantia* adsorbed to PEG microspheres was associated with a significant reduction in the viscosity of hyperglycemic whole blood.

The development of plant-based drugs in nanoscale doses presents a number of advantages, including enhancements of solubility and bioavailability, protection from toxicity, enhancement of pharmacological activity, enhancement of stability, improving tissue macrophage distribution, sustained delivery, and protection from physical and chemical degradation [45]. High glucose levels impair a series of physiological processes, including blood viscosity. Here, adsorption of *M. charantia* to PEG microspheres was effective; the system increased the effects of the extract of *M. charantia,* with a significant reduction of blood viscosity in diabetic patients, to reach values similar to normoglycemic individuals.

The clinical potential for the use of PEG microsphere adsorbed with *M. charantia* [25] and other plants [23, 24] has been reported. Studies show that the adsorption of plants to the PEG microsphere, independent of blood glucose levels, is able to exert immunostimulatory effects on blood cells, activating prooxidative mechanisms as well as the functional activity of these cells. This study is the first to demonstrate the effects of plant-adsorbed polymer systems on blood viscosity. The extract of *M. charantia* may be related to the structural and functional recovery of cells deficient in blood tissue, which may improve the flow characteristics, as glycemic control, especially when the vehicle is PEG microspheres.

The literature also reports that, in addition to diabetes, rheological parameters are used in cardiovascular research, with value for the detection of patients who are prone to cardiovascular disease, monitoring women at risk of pre-eclampsia, assessing hydration status in patients prior to undergoing surgery or catheterization, and monitoring blood viscosity at low temperatures such as in bypass surgery. In addition, its use will facilitate the development of new drugs able to specifically reduce whole blood viscosity by influencing factors such as red blood cell deformability [45].

## 5. Conclusion

These data suggest that the effects of diabetes on the viscosity of the blood should be considered. The use of nanofraction extracts of *M. charantia* and its adsorption on PEG microspheres may represent an alternative for the control and treatment of blood disorders in diabetic patients.

## Figures and Tables

**Table 1:** General and hematological characteristics of the experimental groups.

Parameters	Normoglycemic	Hyperglycemic	Statistics
Age (year)	30.25 ± 9.67	55.00 ± 9.10	*P* > 0.05
Stature (m)	1.68 ± 0.06	1.69 ± 0.06	*P* > 0.05
Body mass (kg)	72.25 ± 21.92	76.10 ± 21.83	*P* > 0.05
Abdominal circumference (cm)	86.00 ± 15.47	99.30 ± 12.02	*P* > 0.05
Body mass index (BMI)	25.63 ± 6.37	26.76 ± 6.07	*P* > 0.05
Erythrocytes (10^6^ *μ*L)	5.23 ± 0.28	4.58 ± 0.36	*P* > 0.05
Leukocytes (10^6^ cells mL^−1^)	5.15 ± 0.84	5.75 ± 1.63	*P* > 0.05
Glycemia (mg dL^−1^)	94.50 ± 9.74	232.80 ± 95.12	*P* = 0.007*

Data are represented by mean ± standard deviation (SD). *Statistical differences (*P* < 0.05) between the normoglycemic and hyperglycemic groups, considering the samples.

**Table 2:** Viscosity of whole blood in the different groups.

Experimental group	Viscosity (Pa·s × 10^−3^)		Statistics
	Normoglycemic	Hyperglycemic	
Whole blood (WB)	3.84 ± 2.80	4.28 ± 3.19	*P* = 0.0008*
WB *M. charantia L*.	2.87 ± 2.68	2.91 ± 2.88	*P* = 0.0511**
WB PEG microsphere	2.94 ± 2.48	3.08 ± 2.42	*P* = 0.0437*
WB PEG *M. charantia L*.	2.98 ± 3.01	2.69 ± 2.58	*P* = 0.0092*

Data are represented by mean ± standard deviation (SD). *Differences (*P* < 0.05) between the normoglycemic and hyperglycemic groups, considering the samples. **Tendency to significance (0.05 < *P* < 0.10) between the normoglycemic and hyperglycemic groups, considering the samples.
